# Selenium Yeast Dietary Supplement Affects Rumen Bacterial Population Dynamics and Fermentation Parameters of Tibetan Sheep (*Ovis aries*) in Alpine Meadow

**DOI:** 10.3389/fmicb.2021.663945

**Published:** 2021-07-02

**Authors:** Xiongxiong Cui, Zhaofeng Wang, Yuhui Tan, Shenghua Chang, Huiru Zheng, Haiying Wang, Tianhai Yan, Tsedan Guru, Fujiang Hou

**Affiliations:** ^1^State Key Laboratory of Grassland Agro-ecosystems, Key Laboratory of Grassland Livestock Industry Innovation, Ministry of Agriculture and Rural Affairs, College of Pastoral Agriculture Science and Technology, Lanzhou University, Lanzhou, China; ^2^School of Computing, Ulster University, Antrim, United Kingdom; ^3^Sustainable Agri-Food Sciences Division, Agriculture Branch, Agri-Food and Biosciences Institute, Hillsborough, United Kingdom; ^4^Animal Husbandry Science and Technology Demonstration Park of Maqu County, Gannan, China

**Keywords:** Tibetan sheep, selenium, Qinghai-Tibet plateau, bacterial communities, high-throughput sequencing, grazing, alpine meadow selenium, rumen bacterial communities

## Abstract

Selenium (Se) deficiency is a widespread and seasonally chronic phenomenon observed in Tibetan sheep (*Ovis aries*) traditionally grazed on the Qinghai–Tibet Plateau (QTP). Effects of the dietary addition of Se-enriched yeast (SeY) on the bacterial community in sheep rumen and rumen fermentation were evaluated with the aim of gaining a better understanding of the rumen prokaryotic community. Twenty-four yearling Tibetan rams [initial average body weight (BW) of 31.0 ± 0.64 kg] were randomly divided into four treatment groups, namely, control (CK), low Se (L), medium Se (M), and high Se (H). Each group comprised six rams and was fed a basic diet of fresh forage cut from the alpine meadow, to which SeY was added at prescribed dose rates. This feed trial was conducted for over 35 days. On the final day, rumen fluid was collected using a transesophageal sampler for analyzing rumen pH, NH_3_-N content, volatile fatty acid (VFA) level, and the rumen microbial community. Our analyses showed that NH_3_-N, total VFA, and propionate concentrations in the M group were significantly higher than in the other groups (*P* < 0.05). Both the principal coordinates analysis (PCoA) and the analysis of similarities revealed that the bacterial population structure of rumen differed among the four groups. The predominant rumen bacterial phyla were found to be Bacteroidetes and Firmicutes, and the three dominant genera in all the samples across all treatments were *Christensenellaceae* R7 group, *Rikenellaceae* RC9 gut group, and *Prevotella* 1. The relative abundances of *Prevotella* 1, *Rikenellaceae* RC9 gut group, *Ruminococcus* 2, *Lachnospiraceae* XPB1014 group, *Carnobacterium*, and Hafnia-*Obesumbacterium* were found to differ significantly among the four treatment groups (*P* < 0.05). Moreover, Tax4fun metagenome estimation revealed that gene functions and metabolic pathways associated with carbohydrate and other amino acids were overexpressed in the rumen microbiota of SeY-supplemented sheep. To conclude, SeY significantly affects the abundance of rumen bacteria and ultimately affects the rumen microbial fermentation.

## Introduction

Rumen, a digestive organ that differentiates uminants from other mammals, is the most efficient natural fermentation system. In this organ, microorganisms ferment and degrade forage fibers and convert them into digestible proteins and volatile fatty acids (VFAs, the primary energy source for ruminants) for digestion and absorption (Dai et al., 2015). Rumen microorganisms are vital for ruminant nutrition as they are directly associated with the animal's diet (Puniya et al., 2015). The rumen microbial community predominantly consists of bacteria, in addition to fungi, protozoa, and a small number of phages (Miller et al., 2012), with each microorganism in a dynamic balance of competition and coordination. Several studies have shown that rumen microorganisms are closely linked to the ruminant livestock production efficiency and provide the host with as much as 65–75% of its energy requirement through anaerobic fermentation (Jewell et al., 2015; Chen et al., 2020). The rumen microbial community structure is affected by factors such as host type, health, and diet, and consequently, it varies across different regions and seasons (Yáñez-Ruiz et al., 2015).

In case the diet is nutritionally insufficient, supplying the rumen microorganisms with nutritional supplements is an appropriate practice to improve the feed conversion efficiency. Pino and Heinrichs (2016) reported that dietary minerals affect the rumen microorganism activity by affecting the rate of dilution of rumen digesta and eventually the rumen osmotic pressure. Gut health, trace element nutrition, and gut microbiome community structure are closely interdependent (Zhang et al., 2015; Faulkner et al., 2017; Biscarini et al., 2018; Ishaq et al., 2019). Research on this complex relationship between microelements and rumen microorganisms is ongoing since many decades. Selenium (Se) is an essential microelement for all life forms and plays a crucial role in livestock productivity (Białek and Czauderna, 2019). Several studies have examined the role of Se administration in improving the rumen efficiency and livestock production. Results of both *in vivo* and *in vitro* culture experiments have shown that Se supplementation promotes the rumen microorganism populations and improves the rumen's digestive efficiency (Shi et al., 2011). Naziroglu et al. (1997) reported that 0.3 mg/kg of Se simultaneously and effectively stimulates the production of total volatile fatty acid (TVFA), acetate, propionate, and butyrate in the rumen. A study by Miltko et al. (2016) reported that the administration of Se-enriched yeast (SeY) supplement increases the production of straight-chain and iso-branched-chain VFA in the rumen of Corriedale lambs. Morsy et al. (2019) evaluated the effect of oral administration of SeY in combination with vitamin E (SYPE) on rumen fermentation in Barki goats and observed an increase in the production of short-chain fatty acids, particularly of propionic acid. Another study showed that both selenized yeast and selenate increase the levels of acetate, propionate, butyrate, valerate, isobutyrate, and isovalerate in the rumen (Białek and Czauderna, 2019). Therefore, organic Se in the form of SeY has a good application prospect because it is safer and more efficient than inorganic Se (Kišidayová et al., 2014). Previous studies have shown that Se plays a role in improving the rumen function; however, its role in rumen microbial community metabolism remains poorly understood. Supplementation of sheep with sodium selenite or selenized yeast affects the population structure of rumen ciliate protozoa, with *Ophryoscolex* being the most Se-sensitive genus (Mihaliková et al., 2005). Although studies have been published on the biochemistry of ruminant Se nutrition, literature on the associated microbiological aspects is scarce.

The Qinghai–Tibet Plateau (QTP), with an average altitude of 4,500 m, is the world's highest plateau that accounts for one-quarter of China's landmass and offers one of the most extreme environments for livestock grazing (Thompson et al., 2000). The cold temperature- and hypoxia-resistant Tibetan sheep (*Ovis aries*) are the main source of income for local herders, providing them with precious resources, such as milk, meat, and fur and wool clothing, and dung as fertilizer and fuel (Zhao et al., 2011). The current population of ~50 million Tibetan sheep extensively grazes in alpine meadows, ranging in altitude between 3,000 and 5,000 m (Zhou et al., 2015). The rumen microflora of the Tibetan sheep are affected by the harsh high-altitude environment, host type, age, and diet, and a greater percentage of novel species belongs to the phylum Firmicutes than that found in the rumen of lowland sheep (Huang et al., 2017). Microflora community comparisons between Tibetan sheep and Yak revealed that the α-diversity indices for the Tibetan sheep are significantly lower than those for Yak, with the rumen fungal community being affected by the host type (Guo et al., 2020). Han et al. (2020) reported that the rumen bacterial community of adult ewes is more diverse than that of rams and yearling ewes. Rumen bacterial diversity analysis of the Tibetan sheep through sequencing indicated the predominance of the phylum Firmicutes, with the remaining sequences belonging to Bacteroidetes, Proteobacteria, Actinobacteria, and unclassified operational taxonomic units (OTUs) (Huang et al., 2017). In our previous study, Bacteroidetes and Firmicutes were identified as the predominant rumen microbial phyla in the yearling Tibetan sheep, whereas the rumen of oat hay fed sheep was found to have a higher proportion of Proteobacteria and novel bacteria than the native pasture-fed sheep (Cui et al., 2019).

The Se content in plant-based diet of herbivore can vary remarkably across different regions (Surai and Fisinin, 2014). The soils of many regions of the world are low in Se; therefore, the majority of grazing animals in these areas receive insufficient dietary Se for their optimum health and performance, leading occasionally to clinical Se deficiency (Schrauzer and Surai, 2009). More than two-thirds of China's pastoral land is Se deficient, and hence, grazing livestock are unable to meet their dietary Se requirement from forage alone (Tashi et al., 2005; Xia et al., 2005). Research have shown that Se deficiency is widespread in Yak and Tibetan sheep of the QTP, often resulting in muscle atrophy, white muscle disease, and loss of animal production (Standing Committee on Agriculture, SCA, 1990; Xiong et al., 2018). Hence, correct feeding and management are essential to ensure the health and productivity of Tibetan sheep. Yet Tibetan herders typically follow the traditional grazing management model, in which the sheep continuously graze all year-round without Se supplementation. Moreover, the effect of Se deficiency on ruminants has received little attention so far. Although our previous study showed that SeY could benefit Tibetan sheep by improving nitrogen metabolism and nutrient digestibility (Wang et al., 2019), the effect of Se dose rates on rumen fermentation parameters and microbial communities is still unknown.

Considering these Se deficiency-related livestock health and performance problems in the QTP, in the present study, we evaluated the effects of Se dietary supplement administered as SeY on rumen fermentation and bacterial community structure. The results of this study will broaden our understanding of rumen bacterial diversity and provide a scientific reference for determining appropriate SeY supplement dose rates to improve the Tibetan sheep production.

## Materials and Methods

### Diet and Livestock Management

Feeding trials were conducted over a 35-day period from July to September (i.e., during warm season) at the Animal Husbandry Science and Technology Demonstration Park, Maqu County (N35° 58′, E101° 53′), Gansu Province, China. Twenty-four yearling Tibetan rams in good body condition [mean body weight (BW): 31.0 ± 0.64 kg] were selected from local pasture as test animals. These were divided randomly into four treatment groups, each of six rams, and fed a basic diet of fresh forage cut from alpine meadow, to which SeY was added at prescribed dose rates: control [CK: 0.00 g SeY/kg dry matter (DM)], low Se (L: 0.09g SeY/kg DM), medium Se (M: 0.18g SeY/kg DM), and high Se (H: 0.36g SeY/kg DM); taking into account the Se contents of the native pasture, the actual Se contents of the four diets were providing 0.023, 0.223, 0.423, and 0.823 mg Se/kg forage DM, respectively.

The trial period included 7 days in penned groups for acclimation and 28 days of formal experimentation. Each sheep was fed in a separate cage, with free access to water, and freshly cut native pasture was fed at 9:00 a.m., 11:00 a.m., 2:00 p.m., and 5:00 p.m. Each day, the forage being chopped into 5- to 10-cm lengths. Dominant pasture families were *Cyperaceae* (24.4%), *Poaceae* (18.6%), *Ranunculaceae* (17.8%), *Compositae* (17.2%), *Liliaceae* (4.1%), and *Fabaceae* (3.1%). SeY supplement (Angel Yeast Co., Ltd., of Hubei, China) was added evenly to this forage and fed once a day at 8:30 a.m. The chemical contents of native pasture and SeY specific parameters are listed in Table 1.

**Table 1:** The chemical contents of diets [as dry matter (DM) basis] and selenium-enriched yeast (SeY) specific parameters.

**Diets**		**SeY**	
DM	35.8%	Appearance properties	Light yellow powder
Organic matter	89.9%	Selenium content	0.2%
Crude protein	7.3%	Drying weightlessness	≤6%
Neutral detergent fiber	59.3%	Ash	≤9%
Acid detergent fiber	32.7%	Relevant substances	≥85%
Ether extract	3.0%	Heavy metals	<3 ppm
Ash	10.0%		
Selenium (mg/kg)	0.023		

The Ethics and Animal Welfare Committee of Lanzhou University authorized all animal experimental procedures (file no: 2012-1 and 2012-2).

### Sample Collection

On the final day of the experiment, and 2 h after feeding, a 10-ml sample of rumen fluid was obtained from each sheep using a transesophageal sampler, after first discarding the initial 50 ml to minimize contamination by oral saliva. Each sample was immediately sealed in a centrifuge tube and frozen at −20°C for subsequent analysis.

### Analysis of Rumen Fermentation Products

The pH of rumen fluid samples was measured using a digital PB-21 pH meter (Sartorius, Germany). NH_3_-N concentration was determined according to a colorimetric method using visible-light spectrophotometry (Agilent Cary 60 UV-Vis Spectrophotometer, USA). Volatile fatty acids were separated and quantified by gas chromatography (Focus GC AI 3000 Thermo Finnigan analyzer). One-microliter preprocessed sample was injected using a split (20:1) and using a Thermo Scientific AI 3000 autosampler (USA) on a Thermo Scientific TRACE 1300 Gas Chromatograph (USA). Injector and detector temperatures were both set at 200°C. Initial column temperature was 45°C, and the rate of temperature rise was set at 20°C/min. At 150°C, column temperature was held for 8 min, followed by further heating at 60°C rise/min until 185°C, where it was held for 1 min. The carrier gas used was nitrogen (purity: 99.99%; flow rate: 40.0 ml N_2_/min).

### DNA Extraction, Polymerase Chain Reaction Amplification, and Sequencing

Each sample was thawed separately and centrifuged at 4°C, and then 200 μl of the supernatant was taken for further analysis. CTAB method was used to extract total rumen microbial genomic DNA. Atomic spectrophotometry and 1% agarose gel electrophoresis were used to evaluate DNA concentration and purity. The universal prokaryote primers F336 and 806R were used to amplify the V3–V4 region of 16S rRNA. Polymerase chain reaction (PCR) amplification was carried out in a 20-μl reaction system. The PCR procedure consists of an initial step at 95°C for 2 min, followed by 30 cycles (95°C for 30 s, 55°C for 30 s, 72°C for 30 s), and a final extension at 72°C for 5 min. PCR expansion products of each sample were then mixed and detected by 2% agarose gel electrophoresis. Each PCR sample had two replicates for pyrosequencing. Sequencing of each PCR product (~200 ng) was done by Novogene Bioinformatics Technology (Beijing, China) using the lon S5^™^ XL platform.

### Sequence Analysis

Raw pyrosequencing single-end reads (400/600 bp) were assigned to samples sorted with barcodes and quality trimmed by FLASH 1.2.7 and QIIME 1.8.0 (Caporaso et al., 2010). The raw reads were spliced and filtered to obtain the high-quality clean reads following the quality controlled procedure and referenced against the SILVA database (Edgar et al., 2011; Martin, 2011; Quast et al., 2012). Sequence analyses was clustered by UPARSE into OTUs based on 97% similarity (Edgar, 2013). A representative sequence for each OTU was screened, based on the mothur algorithm and using the SILVA database to further annotate taxonomic information (Quast et al., 2012). To identify the dominant species within each sample and determine the phylogenetic relationships between OTUs, taxonomic information was obtained, and the number of communities present in each sample was counted at each level of classification. Multiple sequence alignment was conducted and normalized using MUSCLE 3.8.31 (Edgar, 2004).

### Statistical Analysis

For each bacterial community sample, species (OTU) richness and diversity were estimated using the ACE (abundance based coverage estimator), Sobs (observed species richness), and Chao 1 (species richness) indices; Shannon and Simpson indices (species diversity); and Good's coverage index (sequencing depth). Bacterial community structure of each group was then visualized by principal coordinates analysis (PCoA) based on Bray–Curtis dissimilarity matrices using R software (version 2.15.3). An ecologically organized heatmap of the top 35 most abundant genus was created using R software. All these index analyses were based on normalized data, calculated with QIIME 1.8.0 and displayed with R software. Based on the 16S rRNA gene sequence, the Tax4Fun microbial community function prediction used the SILVA database to classify OTU species, query the 16S copy number of each species (OTU) according to its NCBI genome annotation, and standardize the OTUs. Finally, the linear relationship between the SILVA classification and Kyoto Encyclopedia of Genes and Genomes (KEGG) database prokaryotic classification realized the prediction of the microbial community function (Aßhauer et al., 2015). The ruminal fermentation parameters and the α-diversity indices were analyzed using a completely randomized design by one-way analysis of variance (IBM SPSS Statistics 25.0). The Kruskal–Wallis rank sum test was used to compare the bacteria relative abundances between the four treatments using IBM SPSS Statistics 25.0. Significant difference value was set at *P* < 0.05.

## Results

### Rumen Fermentation Parameters

Rumen fermentation parameters are shown in Table 2. NH_3_-N, TVFA, and propionate concentrations in the M group were significantly higher than those in the other groups (*P* < 0.05). Acetate concentration and acetate-to-propionate ratio were both highest in the M group, which also had the lowest isobutyrate concentration (*P* < 0.05). Butyrate concentration was significantly higher in CK group (*P* < 0.05). There was no significant difference between the isovalerate, valerate concentrations, and pH of all four treatment groups (*P* > 0.05).

**Table 2:** Influence of Selenium dose rate on rumen fermentation parameters in Tibetan sheep.

**Rumen fluid analyses**^**2**^	**Treatments**^**1**^				**SEM**	***P-*value**
	**CK**	**L**	**M**	**H**		
pH	6.40	6.39	6.42	6.33	0.05	0.938
NH_3_-N (mg/dl)	17.70^a^	19.01^a^	22.49^b^	18.66^a^	0.18	0.001
TVFA (mmol/L)	29.74^a^	30.26^a^	32.87^b^	31.23^b^	0.35	0.001
Acetate (%)	68.19^a^	68.12^a^	68.21^a^	69.73^b^	0.23	0.02
Propionate (%)	18.64^a^	19.74^b^	20.05^b^	18.41^a^	0.20	0.001
Isobutyrate (%)	2.11^a^	2.10^a^	2.00^a^	1.87^b^	0.34	0.046
Butyrate (%)	5.78^a^	6.30^b^	6.60^b^	7.18^c^	0.10	0.001
Isovalerate (%)	1.88	1.95	1.92	1.87	0.06	0.97
Valerate (%)	0.58	0.54	0.54	0.54	0.01	0.545
Acetate/propionate	3.66^a^	3.46^b^	3.41^b^	3.79^a^	0.04	0.001

1 *CK, L, M, and H: treatment groups supplemented with SeY at 0 (control), 0.09 (low), 0.18 (medium), and 0.36 (high) g/kg dry matter, respectively*.

2 *NH_3_-N: ammonia nitrogen; TVFA: total volatile fatty acids*.

a,b,c *Within rows, means without a common superscript differ (P ≤ 0.05)*.

### Bacterial Species Diversity

16S rRNA gene sequencing of all rumen fluid samples generated a total of 1,831,848 high-quality sequences with an average of 76,327 ± 1,517 [mean ± standard deviation (*SD*), *n* = 24] per sample. Nucleotide sequence identity was divided between reads into separate OTUs, based on a 97% similarity threshold, to obtain 5,032 OTUs. The sample rarefaction curves in Supplementary Figure 1 reached the plateau period, indicating that the sequencing volume is sufficient to contain most of the microbial information in the sample. From the Good's coverage index, coverage of each sample reached 0.99, which reflected that sampling quality was sufficient in all the samples. Table 3 shows that there was no significant difference in species diversity between all four treatment groups (*P* > 0.05).

**Table 3:** Operational taxonomic unit count and diversity estimation from sequencing analysis and based on the 16S rRNA gene libraries.

**Item**	**Treatments**^**1**^				**SEM**	***P*-value**
	**CK**	**L**	**M**	**H**		
Observed species	1,341	1,332	1,341	1,321	15.03	0.249
Shannon	7.16	7.62	7.29	7.42	0.08	0.189
Simpson	0.96	0.98	0.97	0.97	0.01	0.277
Chao 1	1,571	1,543	1,578	1,575	21.16	0.942
ACE	1,603	1,556	1,589	1,600	18.52	0.818
Good's coverage	0.99	0.99	0.99	0.99	0.00	0.192

1 *CK, L, M, and H: treatment groups supplemented with SeY at 0 (control), 0.09 (low), 0.18 (medium), and 0.36 (high) g/kg dry matter, respectively*.

### Comparison of Bacterial Community Composition and OTUs Between Treatments

**Figure 1:**
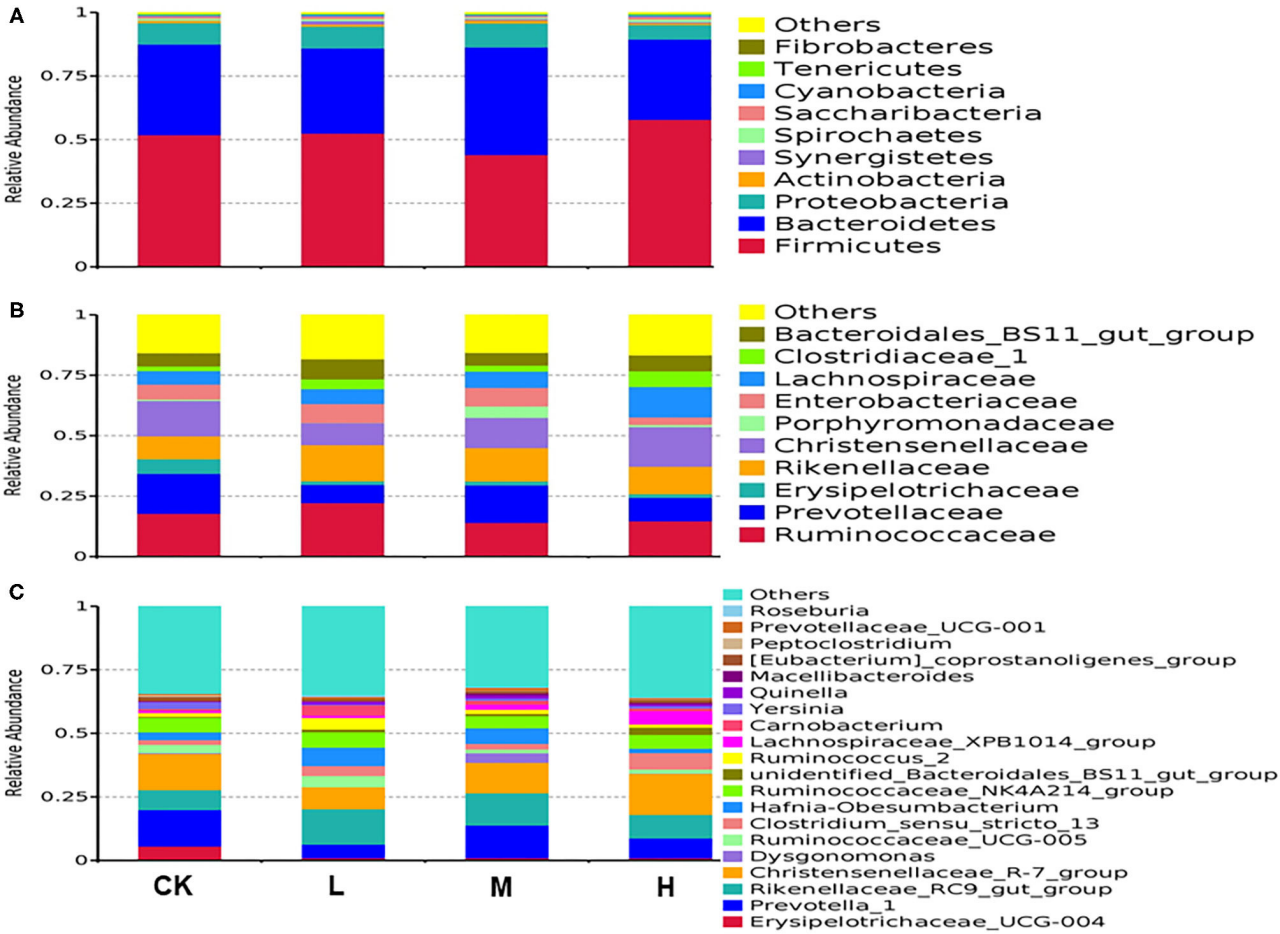
Relative abundance of operational taxonomic units agglomerated of bacterial taxa averaged under phylum level for **(A)**, family level for **(B)**, and genera level for **(C)** at the four SeY doses (mg SeY/kg dry matter) (CK, 0; L, 0.09; M, 0.18; H, 0.36). The color-coded bar plot shows the average bacterial phylum, family, and genus distributions level of the nutritional diets sampled. CK, control; L, low Se, M, medium Se; H, high Se.

Rumen fluid flora analyses indicated that 23 phyla were present in all samples. The predominant phyla in all four treatment groups according to phylum assignment results are listed in Supplementary Table 1. The most abundant phylum was Firmicutes (CK 51.88%, L 52.51%, M 44.09%, and H 57.89%, respectively), the secondary phylum, Bacteroidetes (CK 35.64%, L 33.41%, M 42.25%, and H 31.57%, respectively), and the tertiary phylum, Proteobacteria (CK 8.36%, L 8.65%, M 9.42%, and H 5.69%, respectively) (Figure 1A). The most differentially abundant bacterial phylum in the four groups was Synergistetes (CK 0.14%, L 1.34%, M 0.55%, and H 0.45%, respectively), and the relative abundance of Synergistetes decreased with SeY additive level (*P* < 0.05) (Supplementary Table 1). At the family level, *Ruminococcaceae* (17.22%), *Christensenellaceae* (13.13%), *Rikenellaceae* (12.38%), and *Prevotellaceae* (12.29%) were the dominant families; other families included *Lachnospiraceae* (7.74%), *Bacteroidales* BS11 gut group (6.43%), *Enterobacteriaceae* (6.20%), *Clostridiaceae* 1 (3.19%), *Erysipelotrichaceae* (2.72%), and *Porphyromonadaceae* (1.60%) (Figure 1B).The difference in relative abundances of *Lachnospiraceae* was statistically significant (*P* < 0.05) (Supplementary Table 2). At the genus level, 274 genera were detected in all the samples, of which the 15 most abundant genera were found in the four groups (average relative abundance of >1% for at least one group) in the Tibetan sheep rumen, presented in Supplementary Table 3. The three dominant genera in all groups were *Christensenellaceae* R7 group (CK 14.28%, L 8.71%, M 11.98 %, and H 16.16%, respectively), *Rikenellaceae* RC9 gut group (CK 7.8%, L 13.95%, M 12.72%, and H 9.2%, respectively), and *Prevotella* 1 (CK 14.35%, L 5.29%, M 12.84%, and H 7.9%, respectively) (Figure 1C).

**Figure 2:**
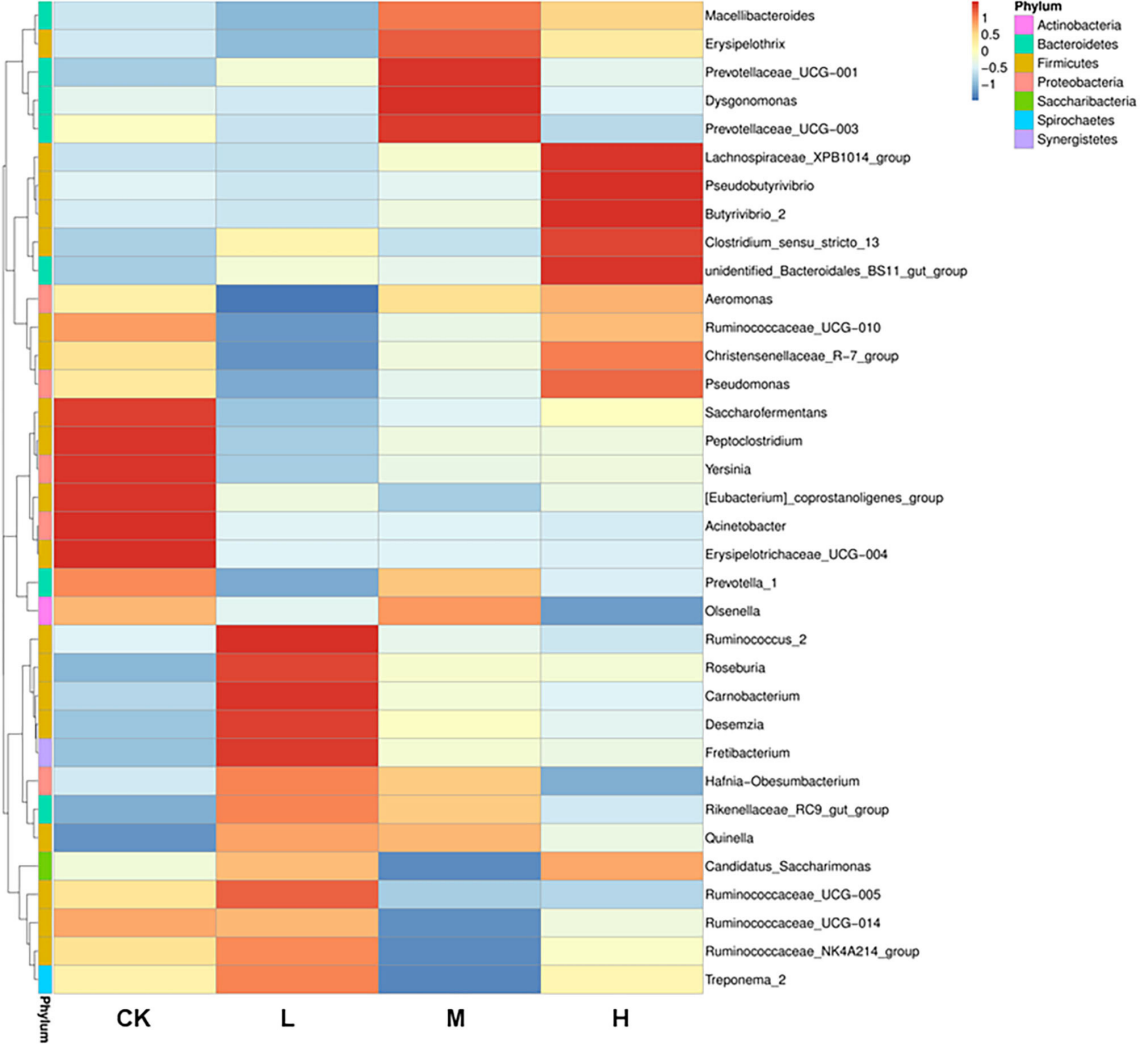
Heatmap analysis of 35 shared prokaryotic community genera of all Tibetan sheep, as determined by the relative abundance of each shared genera through sequencing platform. The vertical direction is the sample information, and the horizontal direction is the species annotation information. The left cluster tree is the species clustering tree; the middle heatmap corresponds to the value of each row of the relative abundance of species after the standardized treatment of the *Z*-score.

**Figure 3:**
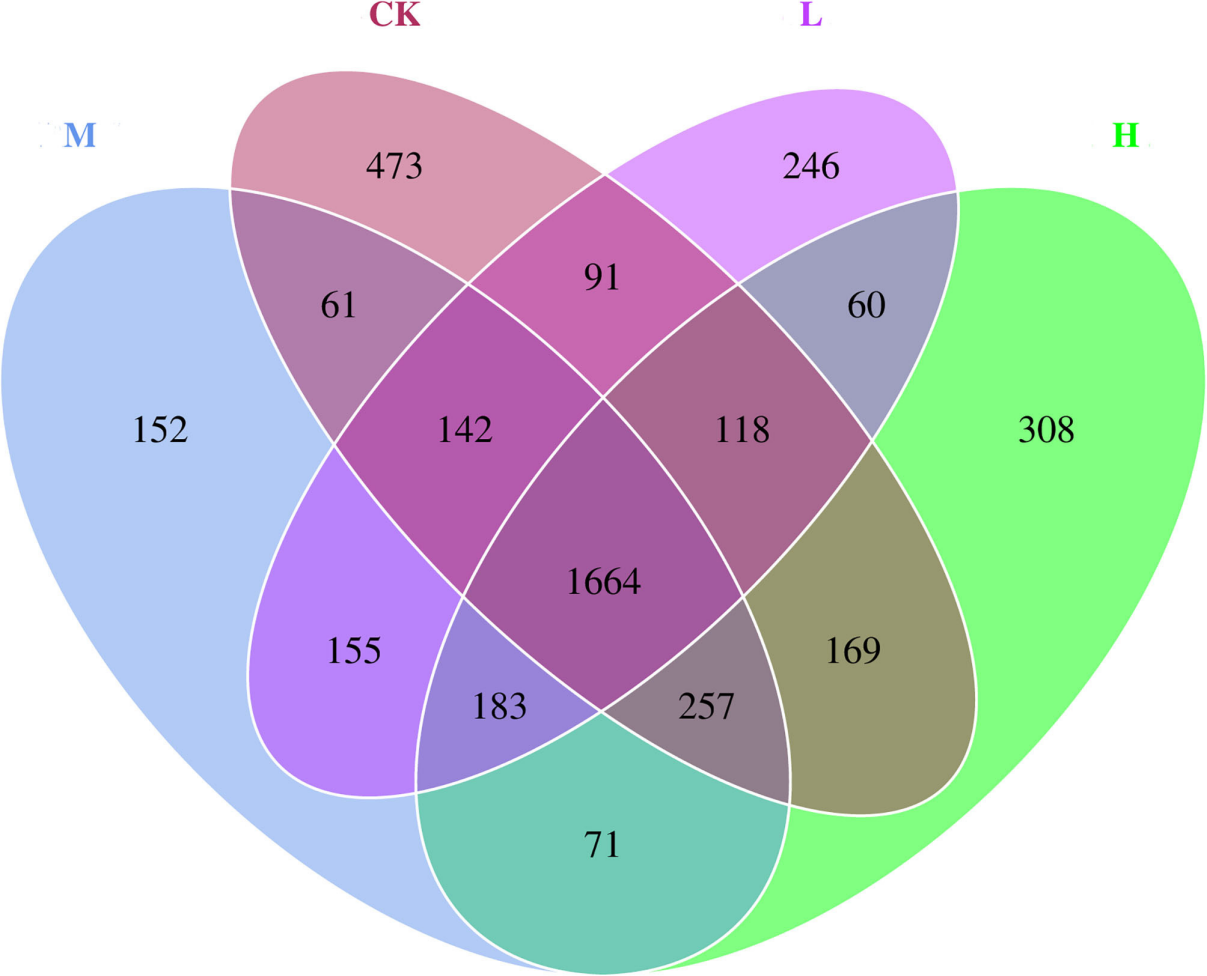
Microbial diversity Venn diagram of rumen bacteria of Tibetan sheep at the four SeY doses (mg SeY/kg dry matter) (CK, 0; L, 0.09; M, 0.18; H, 0.36). CK, control; L, low Se, M, medium Se; H, high Se.

The differences in relative abundance of *Christensenellaceae* R-7 group, *Ruminococcus* 2, *Lachnospiraceae XPB1014 group, Carnobacterium, Prevotella 1, Dysgonomonas*, and Hafnia-*Obesumbacterium* in various groups were statistically significant (*P* < 0.05) (Supplementary Table 3). In order to provide clarity and visualization, a heatmap showed the top 35 genera (Figure 2). Based on heatmap, the results showed that the abundances of *Saccharofermentans, Peptoclostridium, Yersinia* [Eubacterium] *coprostanoligenes* group, *Acinetobacter*, and *Erysipelotrichaceae* UCG-004 were higher in the CK group than in the groups receiving Se supplement. However, the low-level Se (L group) intake correlated positively with the abundance of *Ruminococcus* 2, *Roseburia, Carnobacterium, Desemzia, Fretibacterium*, and *Ruminococcaceae* UCG-005; the medium level Se (M group) intake correlated positively with the abundance of *Prevotellaceae* UCG-001, *Dysgonomonas*, and *Prevotellaceae* UCG-003; and the high-level Se (H group) intake correlated positively with the abundance of *Lachnospiraceae* XPB1014 group, *Pseudomonas, Butyrivibrio* 2, and unidentified *Bacteroidales* BS11 gut group. In all samples, a total of 4,150 OTUs were calculated. The number of OTUs shared among the four groups was 1,664, and the number of OTUs specific to the CK, L, M, and H groups was 473, 246, 152, and 308, respectively (Figure 3).

### Se and Rumen Bacterial Community Dynamics

**Figure 4:**
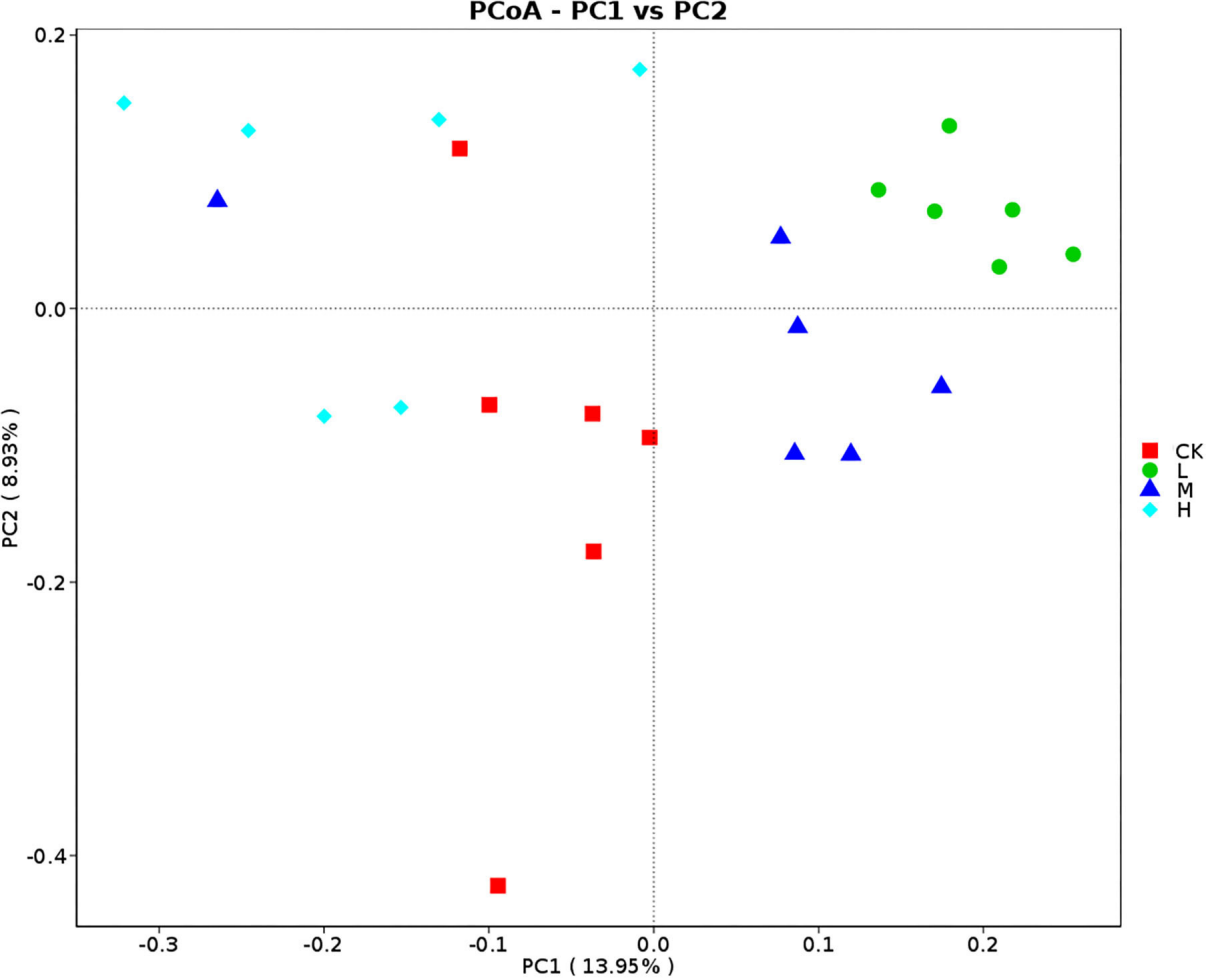
PCoA (Bray–Curtis) of rumen bacteria in Tibetan sheep at the four SeY doses (mg SeY/kg dry matter) (CK, 0; L, 0.09; M, 0.18; H, 0.36). CK, control; L, low Se, M, medium Se; H, high Se.

Principal coordinates analysis analyses based on Bray–Curtis dissimilarity matrices showed that rumen bacterial communities at four groups gathered together by their ration treatment and separated (Figure 4). The results of analysis of similarities strengthen the tendency of the difference of community structure of the four groups (CK-L: *P* = 0.005; CK-M: *P* = 0.038; CK-H: *P* = 0.009; L-M: *P* = 0.029; L-H: *P* = 0.004; M-H: *P* = 0.015).

### Tax4Fun Prediction of Functional and Metabolic Capabilities

**Figure 5:**
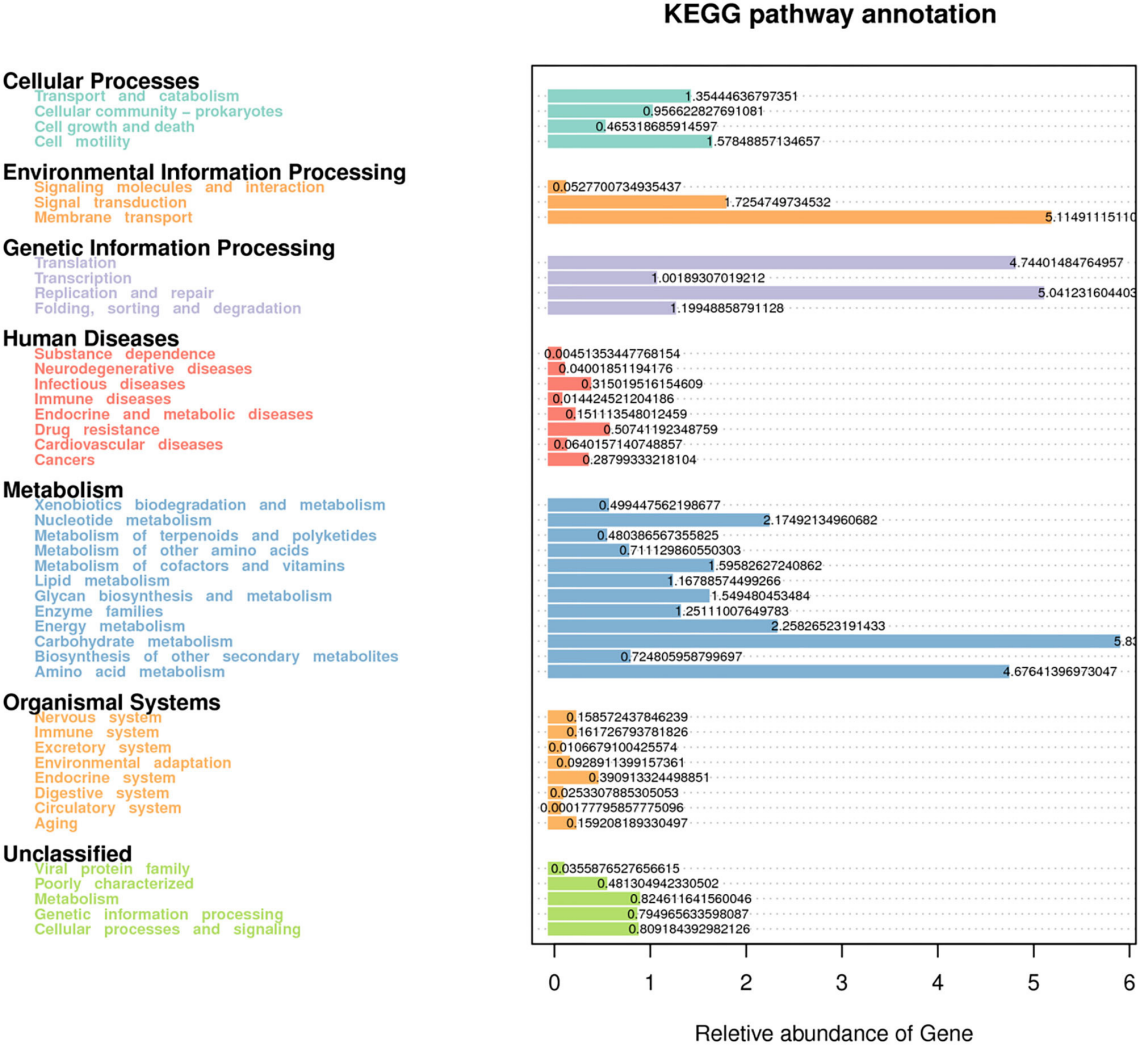
Predictive functional analysis using Tax4Fun tool, showing relative abundance of assigned KEGG identity to the pathways of metabolisms at subsystem levels (KO_2_).

Forty-four functional pathways were identified based on the predicted metagenomes at the level 2 KEGG orthologs (KO) and their corresponding pathways (Figure 5). In this functional profile, the overall functional structure of the bacterial community identified using rumen fluid samples was found to be dominated by the following KEGG pathway-related processes: metabolism (carbohydrates, amino acids, nucleotides, and energy); genetic information processing (translation, replication, and repair); and environmental information processing (membrane transport and signal transduction). Regarding cellular processes in prokaryotes, the genes responsible for cellular motility and transport and catabolism pathways were found to be dominant. The genes related to human diseases and organismal system pathways were also predicted. Other KEGG pathways in the inferred functional profile of bacterial communities in rumen fluid could not be classified, although these pathways were principally related to metabolism, genetic information processing, and cellular processes and signaling. Eleven pathways showed significant differences among the CK, L, M, and H groups (*P* < 0.05) (Supplementary Table 4).

## Discussion

Biodiversity metrics evaluate the sustainability and productivity within numerous ecosystems, and the diversity of the gut intestinal microbiota is closely related to health, metabolic ability, and stability of animals (Fan et al., 2020). In this study, the bacteria diversity index of Tibetan sheep was not found to vary significantly between the groups receiving SeY supplement and the CK group, suggesting that Se-supplemented diets do not affect the diversity of rumen bacterial community in Tibetan sheep. A study on growing Beagle puppies reported that the α-diversity index of the gut bacterial community is not affected by the Se source (sodium selenite or SeY) (Pereira et al., 2020), which is consistent with our results.

Dietary changes play an important role in altering the composition of rumen microbial communities. However, regardless of the diet composition, Firmicutes and Bacteroidetes were reported to be the dominant phyla in the rumen (Jami et al., 2013), indicating that these bacteria play an important role in the rumen function and ecology of ruminants. In this study, the dominant phyla were Firmicutes and Bacteroidetes, and the finding is consistent with those of several studies on herbivores (Rubino et al., 2017; Cui et al., 2019). The dominant microbial community in the rumen of ruminants is not static and may be adjusted dynamically based on the conventional dominant bacterial group with changes in the feed type (McCann et al., 2016). Therefore, the investigation of rumen microorganisms is usually performed under specific dietary conditions (Clemmons et al., 2019). A previous study showed that members of the Bacteroidetes phylum are mainly responsible for protein hydrolysis and carbohydrate degradation, whereas those belonging to the phylum Firmicutes play an important role in energy utilization (Wu et al., 2011; Chen et al., 2015). In this study, we identified that the diet supplemented with a medium level of SeY may be more suitable for the survival of the phylum Bacteroidetes, whereas a high level of SeY may be more suitable for the survival of the phylum Firmicutes. The trend of increase in the relative abundance of Bacteroides and Bacteroidetes/Firmicutes indicated that a medium level of SeY may improve the protein and carbohydrate utilization rate of native pasture. Interestingly, when SeY was included as a dietary supplement, the abundance of Synergistetes (an uncommon bacterial phylum) was influenced by the increasing dietary SeY-supplement level. Synergistetes are found under diverse anaerobic environments including soils, wastewater treatment systems, and human oral cavity, although they are not regular members in the rumen (Godon et al., 2005). According to a report, Synergistetes have diversified by exploiting the same type of metabolic niche under different habitats (Hugenholtz et al., 2010). We speculate that Synergistetes are mainly responsible for Se utilization in the rumen; however, this assumption requires further investigation. At the family level, the relative abundance of *Lachnospiraceae* increased with an increase in the SeY supplementation level. A previous study showed that *Lachnospiraceae* plays an essential role in producing beneficial metabolites for the host, with some members exhibiting strong hydrolyzing activities (Sagheddu et al., 2016). Many *Lachnospiraceae* species are associated with the production of butyrate, which assists in maintaining a healthy ruminant intestinal environment (Louis and Flint, 2009). Meehan and Beiko (2014) showed that *Lachnospiraceae* species are involved in preventing colon cancer. In this feeding trial, the relative abundance of the species belonging to the family *Lachnospiraceae* was found increased with SeY-supplemented level. We speculate that this SeY supplementation-induced response could possibly help Tibetan sheep improve their health and metabolic capability.

Importantly, at the genus level, the abundance of *Christensenellaceae* R7 group, *Ruminococcus* 2, *Lachnospiraceae* XPB1014 group, *Carnobacterium, Prevotella 1, Dysgonomonas*, and *Hafnia-*Obesumbacterium showed clearly distinct responses to dietary Se supplementation; however, the predominant OTU in the rumen microbiome in all the four treatments was the *Christensenellaceae* R7 group. A study showed that *Christensenellaceae* are associated with a lean host phenotype of human (Goodrich et al., 2014). Our previous study reported that dietary supplementation of SeY could improve the food conversion ratio of Tibetan sheep and that there is a quadratic relationship between the SeY supplementation level and food conversion ratio, wherein a high Se dose rate is not beneficial for sheep growth (Wang et al., 2019). *Christensenellaceae* R7 may play an important role in the degradation of hemicellulose and cellulose (Dai et al., 2015). In this study, the abundance of *Christensenellaceae* R7 showed a decreasing to increasing trend with an increase in dietary SeY supplementation. This result supports the hypothesis that dietary supplementation with high amounts of SeY increases the *Christensenellaceae* R7 abundance, which may be disadvantageous in promoting the growth of Tibetan sheep, whereas supplementing the diet with low or medium amounts of SeY may be beneficial in promoting their growth and development. The *Ruminococcaceae* family plays an important role in mucosa associated in colon and cellulose degradation of mammals (Nava and Stappenbeck, 2011). The *Ruminococcus* genus consists of two types of powerful fiber-degrading bacteria that can break down fibers into hemicellulase and cellulose through rumen fermentation, and cellulose can be further degraded to VFAs (Jami and Mizrahi, 2012). Therefore, the abundance of *Ruminococcus* may affect the energy utilization efficiency to support Tibetan sheep adapted to harsh conditions of the QTP environment. In the process of adapting to the low-SeY and medium-SeY diets, the abundance of *Ruminococcus* 2 first increased and eventually decreased in the high-SeY diet group, indicating that SeY supplementation might affect cellulose digestion, VFA production, and energy utilization efficiency. Reduction in these bacterial populations in the high-SeY group might reduce the Tibetan sheep's ability to degrade plant cellulose and energy utilization efficiency. The proportion of the *Lachnospiraceae* XPB 1014 group was higher in the rumen of Tibetan sheep in the high-SeY group than in the rumen of those in other groups, indicating that the genus *Lachnospiraceae* XPB 1014 has different SeY sensitivities. *Carnobacteria* are ubiquitous lactic acid bacteria isolated from different habitats and can catabolize a wide range of carbohydrates. Their importance as probiotic cultures in the food and aquaculture breeding industry has been established (Leisner et al., 2007). A study showed that some *Carnobacterium* members are mainly responsible for humoral and cellular immune responses of various hosts (Kim and Austin, 2006). In the present study, the relative abundance of *Carnobacterium* significantly increased in the rumen of Tibetan sheep that were fed a diet supplemented with medium level of SeY, indicating that such a diet may improve the utilization of dietary carbohydrates and immune capacity. The *Prevotellaceae* family is a metabolically and genetically diverse microbial population in the rumen that possesses the ability to degrade lignocellulosic feedstock (Bi et al., 2018). Moreover, the *Prevotellaceae* family is also involved in pectin and protein metabolism (Schnorr et al., 2014). For ruminants, *Prevotellaceae* plays an important role in degrading oligopeptides (Walker et al., 2003). Proteins as a nitrogen source are essential for the growth of *Prevotella* 1. A correlation analysis revealed that *Prevotella* 1 plays an important role in the metabolism of amino acids in the rumen (Xue et al., 2020). In our study, the abundance of the genus *Prevotella* 1 was found to be affected by SeY-supplemented level. On the basis of the aforementioned experimental results, we conclude that SeY supplementation affects the fermentation of feed, cellulose degradation, and protein metabolism in the rumen of Tibetan sheep and significantly improves when medium amounts of this nutrient are administered as a supplement. This conclusion is supported by the finding of our previous study on digestive and metabolic of Tibetan sheep by SeY supplementation (Wang et al., 2019). Proteobacteria play a crucial role in rumen metabolism, especially in the digestion of soluble carbohydrates, biofilm formation, and fermentation (Pitta et al., 2016). In addition, the abundance of an uncommon potentially pathogenic genus belonging to phylum Proteobacteria, Hafnia-*Obesumbacterium*, in the rumen of Tibetan sheep was found to differ significantly among all the four treatment groups (*P* < 0.05). Some species of Hafnia-*Obesumbacterium* have been detected in frog droppings (Tong et al., 2020), distal intestinal lumen of rainbow trout (Lyons et al., 2017), and human gut (Ramos-Vivas, 2020) but have been rarely reported in rumen fluids of sheep. The relationship between the ruminants and Hafnia-*Obesumbacterium* remains poorly understood and deserves further investigation.

In addition to variations in Tibetan sheep rumen microbiomes, VFA levels varied considerably in response to SeY among all the four treatment groups. Changes in rumen microflora affect VFA production, which eventually affects the efficiency of nutrient utilization (Hernandez-Sanabria et al., 2012). Volatile fatty acid levels produced during rumen fermentation can be considered an important index of rumen fermentation, and the type of rumen fermentation affects the energy utilization rate and energy reserve site (Cone and Becker, 2012). Volatile fatty acids are largely absorbed across the host's ruminal epithelium, which play an essential role in ruminant immunity and growth (Fan et al., 2020). Acetate is the main precursor of milk fat synthesis in ruminants, whereas propionate is an important glucose precursor (Allen, 2014). Thus, propionate-type fermentation is more conducive to the fattening of livestock. In this study, significantly higher levels of propionate and TVFA were observed in the M group (*P* < 0.01). This increase in the propionate concentration resulted in an increase in the TVFA concentration and a decrease in the ratio of acetate to propionate. This result showed that supplementation of Tibetan sheep with a medium level of SeY might lead to more efficient transportation and absorption of VFAs than the CK group. Our previous digestive and metabolic experiments had also shown that a medium Se dose rate (0.4 mg/kg DM) improves nitrogen metabolism and nutrient digestibility, which are beneficial for the growth and development of young Tibetan sheep (Wang et al., 2019). Shi et al. (2011) reported that the addition of 3 g nano-Se/kg forage DM to the basic diet of sheep increases the TVFA concentration in their rumen. A study by Farzaliev (1981) reported that the addition of 0.1 mg Na_2_SeO_3_/kg BW to the high energy diet of young bulls increases VFA and propionate levels and reduces the acetate content. Isoacids (such as isobutyrate and isovalerate) are branched-chain VFAs produced by the oxidative deamination of branched-chain amino acids in the rumen, and the levels of branched-chain VFA correlate with the extent of protein fermentation (Hino and Russell, 1985). These iso-VFAs are required for the normal growth and activity of rumen cellulolytic bacteria (Wolin et al., 1997). In this study, we showed that a high SeY dose rate decreases the iso-VFA levels, suggesting that a high SeY dose inhibits the deamination activity and protein fermentation in the rumen of Tibetan sheep. Rumen bacteria can metabolize inorganic Se by allowing the binding of Se to their own protein. Inorganic Se has been shown to promote rumen bacterial reproduction *in vitro* (Kim et al., 1997). Organic Se used in this study also supports these conclusions. Dietary protein is the main source of rumen NH_3_-N, and its concentration is affected by the degradation and absorption rates of rumen nitrogen, which reflect the rate of nitrogen use by the rumen microorganisms. The present study indicated that SeY supplementation affects the efficiency of conversion of dietary nitrogen into microbial nitrogen that improves significantly when medium doses of SeY are administered, which could be attributable to variations in rumen microflora in the Tibetan sheep with different levels of SeY. This result is supported the finding of our previous study on nitrogen metabolism of Tibetan sheep by SeY supplementation (Wang et al., 2019).

As expected, the prediction analysis of the bacterial community function showed differences among each treatment group. Rumen bacterial communities are capable of adapting to a vast range of ruminant diets, which clearly shows the significance of the rumen microbial ecosystem in providing ruminant nutritional needs. The Tax4Fun prediction tool helped in deriving metabolic pathways at the subsystem level, revealing the potential of SeY use for regulating the rumen microbial population structure and function in Tibetan sheep. Diet usually plays a major role in shaping the rumen microbial community structure. However, to achieve optimum results by dietary intervention, genetic differences among livestock should also be considered (Carmody et al., 2015). Functional prediction analysis revealed that the function of the rumen bacteria varies among the SeY-supplemented groups and the CK group. Our data of Tax4Fun gene function estimation indicate that the relative abundances of carbohydrate metabolism and metabolism of other amino acids were significantly impacted by Se; those genes involved in energy metabolism and carbohydrate were enhanced in supplementing Se in diets of Tibetan sheep. Because of the inherently low nutrients available in partial native pasture, a good production of livestock cannot be guaranteed. Therefore, long-term supplementation of medium levels of SeY is a wise choice for improving the productivity for livestock. Administration of medium levels of Se as a booster to Tibetan sheep grazing on the natural grassland can prevent nutritional deficiencies in these sheep. In addition, SeY may widely affect the interaction among rumen microorganisms, although the mechanism of these interactions remains unclear. Therefore, other scientific methods such as metagenomics analysis are required to further understand the physiological characteristics of the rumen microbes in this species. Notably, we identified a large number of unclassified genera with unknown functions. Our finding suggests that Tibetan sheep may have a highly diverse intestinal microbiome, and further in-depth studies are required to investigate the microbiome diversity.

## Conclusion

The addition of SeY in the diet changed the rumen microflora of Tibetan sheep, which eventually affected the levels of VFAs in the rumen fluid. Abundant variations in the rumen bacteria indicated that the addition of SeY to the diet exerts a positive effect on the rumen microbial community and metabolic function of Tibetan sheep. SeY can be used as a trace element supplement for grazing Tibetan sheep in alpine meadows on the QTP to improve rumen fermentation and microbial community structure. However, considering the risk of Se toxicity, we suggest that the dietary Se level of 0.4 mg/kg DM is optimum. Significant or extremely significant differences in bacterial abundance were observed with variations in SeY doses among different diets administered to the sheep, indicating that these microbial communities might have a specific function in rumen metabolism; however, further studies are required to determine their exact role. Future studies may reveal the characteristics of the microbial composition and population structure in the rumen environment of Tibetan sheep.

## Data Availability Statement

The datasets presented in this study can be found in online repositories. The names of the repository/repositories and accession number(s) can be found below: https://www.ncbi.nlm.nih.gov/, PRJNA627580.

## Ethics Statement

The animal study was reviewed and approved by The Ethics and Animal Welfare Committee of Lanzhou University authorized all animal experimental procedures (file No: 2012-1 and 2012-2).

## Author Contributions

Conceptualization: FH; Original draft preparation: XC; Interpretation participant: HZ, HW, and TY; Study conception and funding: FH; Literature search: ZW, XC, and SC; Experimental work and data collection: XC, YT, and TG; Conception, development, and implementation of computational methods: XC and ZW. All authors contributed to the article and approved the submitted version.

## Conflict of Interest

The authors declare that the research was conducted in the absence of any commercial or financial relationships that could be construed as a potential conflict of interest.
