# Supplementary material for: Selenium Yeast Dietary Supplement Affects Rumen Bacterial Population Dynamics and Fermentation Parameters of Tibetan Sheep (Ovis aries) in Alpine Meadow
Source: Front Microbiol. 2021 Jul 2;12:663945. doi: 10.3389/fmicb.2021.663945 (PMC8283570; doi:10.3389/fmicb.2021.663945)
Supplement: Supplementary file 1 [file Table_1.docx]

**Supplementary Table S1. Relative abundance (%) of bacterial phyla at each dietary treatment (average relative abundance > 0.1% for at least one group).**

| Phylum | Treatments^1^ | | | | SEM | *P*-value |
| --- | --- | --- | --- | --- | --- | --- |
|  | CK | L | M | H |  |  |
| Firmicutes | 51.88 | 52.51 | 44.09 | 57.89 | 1.92 | 0.190 |
| Bacteroidetes | 35.64 | 33.41 | 42.25 | 31.57 | 1.56 | 0.111 |
| Proteobacteria | 8.36 | 8.65 | 9.42 | 5.69 | 0.83 | 0.533 |
| Actinobacteria | 0.91 | 0.78 | 1.17 | 0.70 | 0.15 | 0.485 |
| Synergistetes | 0.14a | 1.34b | 0.55ab | 0.45ab | 0.14 | 0.018 |
| Spirochaetes | 0.82 | 0.82 | 0.67 | 1.05 | 0.1 | 0.640 |
| Saccharibacteria | 0.9 | 1.11 | 0.66 | 1.13 | 0.09 | 0.206 |
| Cyanobacteria | 0.52 | 0.62 | 0.48 | 0.6 | 0.06 | 0.952 |
| Tenericutes | 0.37 | 0.31 | 0.28 | 0.43 | 0.04 | 0.652 |
| Fibrobacteres | 0.39 | 0.39 | 0.41 | 0.43 | 0.03 | 0.213 |
| Others | 0.39 | 0.39 | 0.41 | 0.43 | 0.03 | 0.933 |

^1^CK, L, M, and H, treatment groups supplemented with SeY at 0, 0.09, 0.18, and 0.36 g /kg DM, respectively.

^a,b^Within rows, means without a common superscript differ (*P* ≤ 0.05).

**Supplementary Table S2. Relative abundance (%) of bacterial family at each dietary treatment (average relative abundance >1% for at least one group).**

| Family | Treatments^1^ | | |  | SEM | *P*-value |
| --- | --- | --- | --- | --- | --- | --- |
|  | CK | L | M | H |  |  |
| Ruminococcaceae | 17.85 | 22.26 | 14.06 | 14.71 | 1.39 | 0.141 |
| Prevotellaceae | 16.49 | 7.48 | 15.47 | 9.71 | 1.34 | 0.070 |
| Erysipelotrichaceae | 6.08 | 1.55 | 1.69 | 1.56 | 1.08 | 0.937 |
| Rikenellaceae | 9.42 | 14.97 | 13.82 | 11.29 | 0.95 | 0.176 |
| Christensenellaceae | 14.64 | 9.06 | 12.48 | 16.38 | 1.05 | 0.118 |
| Porphyromonadaceae | 0.61 | 0.13 | 4.74 | 0.93 | 0.84 | 0.079 |
| Enterobacteriaceae | 6.20 | 7.78 | 7.67 | 3.15 | 0.40 | 0.114 |
| Lachnospiraceae | 5.54a | 6.19a | 6.70a | 12.55b | 0.77 | 0.01. |
| Clostridiaceae_1 | 1.97 | 4.04 | 2.48 | 6.48 | 0.70 | 0.075 |
| Bacteroidales_BS11_gut_group | 5.48 | 8.31 | 5.28 | 6.67 | 0.57 | 0.144 |
| Others | 15.72 | 18.24 | 15.61 | 16.58 | 0.79 | 0.537 |

^1^CK, L, M, and H, treatment groups supplemented with SeY at 0, 0.09, 0.18, and 0.36 g /kg DM, respectively.

^a,b^Within rows, means without a common superscript differ (*P* ≤ 0.05).

**Supplementary Table S3. Relative abundance (%) of bacterial genera at each dietary treatment** (**average relative abundance >1% for at least one group).**

| Phylum | Genus | Treatments^1^ | | | | SEM | *P*-value |
| --- | --- | --- | --- | --- | --- | --- | --- |
|  |  | CK | L | M | H |  |  |
| Firmicutes | *Christensenellaceae R-7 group* | 14.28 | 8.71 | 11.98 | 16.16 | 1.04 | 0.035 |
| Firmicutes | *Erysipelotrichaceae UCG-004* | 5.6 | 1.04 | 1.04 | 0.93 | 1.08 | 0.918 |
| Firmicutes | *Ruminococcaceae UCG-005* | 3.23 | 4.37 | 1.52 | 1.63 | 8.67 | 0.631 |
| Firmicutes | *Clostridium sensu stricto 13* | 1.91 | 3.94 | 2.21 | 6.44 | 6.97 | 0.051 |
| Firmicutes | *Ruminococcaceae NK4A214 group* | 5.7 | 6.02 | 4.72 | 5.42 | 0.4 | 0.064 |
| Firmicutes | *Ruminococcus 2* | 1.46 | 4.53 | 1.66 | 1.27 | 0.44 | 0.012 |
| Firmicutes | *Lachnospiraceae XPB1014 group* | 0.97a | 0.92a | 1.99a | 5.2b | 0.49 | 0.013 |
| Firmicutes | *Carnobacterium* | 0.65a | 0.4a | 1.52b | 1.07ab | 0.37 | 0.007 |
| Firmicutes | *[Eubacterium] coprostanoligenes group* | 1.69 | 1.12 | 0.92 | 1.11 | 0.15 | 0.337 |
| Bacteroidetes | *Prevotella 1* | 14.35b | 5.29a | 12.84b | 7.9ab | 1.27 | 0.027 |
| Bacteroidetes | *Rikenellaceae RC9 gut group* | 7.8a | 13.95b | 12.72ab | 9.2ab | 0.92 | 0.079 |
| Bacteroidetes | *unidentified Bacteroidales BS11 gut group* | 0.42 | 1.11 | 0.92 | 2.86 | 0.43 | 0.206 |
| Bacteroidetes | *Dysgonomonas* | 0.34a | 0.01a | 3.72b | 0.14a | 0.79 | 0.035 |
| Proteobacteria | *Hafnia-Obesumbacterium* | 3.04ab | 7.25b | 6.15b | 1.72a | 0.82 | 0.030 |
| Proteobacteria | *Yersinia* | 2.67 | 0.38 | 0.86 | 0.98 | 0.39 | 0.189 |
|  | Others | 28.92 | 30.02 | 26.55 | 28.9 | 0.81 | 0.350 |

^1^CK, L, M, and H, treatment groups supplemented with SeY at 0, 0.09, 0.18, and 0.36 g /kg DM, respectively.

^a,b^Within rows, means without a common superscript differ (*P* ≤ 0.05).

**Supplementary Table S4. Functional predictions for rumen microbiota with significantly different KEGG pathways (Level 2 KOs).**

| KEGG Pathway | | Treatments^1^ | | | | SEM | | *P*-value |
| --- | --- | --- | --- | --- | --- | --- | --- | --- |
|  |  | CK | L | M | H |  |  |  |
| Organismal Systems | Aging | 0.64 | 0.66 | 0.67 | 0.68 | 0.01 | 0.012 | |
|  | Environmental adaptation | 0.40 | 0.40 | 0.38 | 0.37 | 0.01 | 0.007 | |
| Metabolism | Biosynthesis of other secondary metabolites | 2.97 | 2.97 | 3.03 | 3.11 | 0.02 | 0.048 | |
|  | Carbohydrate metabolism | 24.07 | 24.25 | 24.27 | 24.63 | 0.07 | 0.019 | |
|  | Metabolism of other amino acids | 2.91 | 2.92 | 2.97 | 3.04 | 0.02 | 0.021 | |
|  | Metabolism of terpenoids and polyketides | 1.98 | 1.96 | 2.04 | 2.00 | 0.01 | 0.035 | |
| Human Diseases | Cardiovascular diseases | 0.27 | 0.27 | 0.27 | 0.26 | 0.07 | 0.013 | |
|  | Immune system | 0.69 | 0.68 | 0.66 | 0.67 | 0.01 | 0.009 | |
| Unclassified | Poorly characterized | 1.96 | 2.00 | 2.02 | 2.05 | 0.01 | 0.009 | |
| Cellular Processes | Cell motility | 7.05 | 6.91 | 6.58 | 5.76 | 0.17 | 0.017 | |
| Environmental Information Processing | Signaling molecules and interaction | 0.18 | 0.21 | 0.21 | 0.29 | 0.01 | 0.024 | |

^1^CK, L, M, and H, treatment groups supplemented with SeY at 0, 0.09, 0.18, and 0.36 g /kg DM, respectively.

^a,b^Within rows, means without a common superscript differ (*P* ≤ 0.05).
