# Supplementary figures and images for: Selenium Yeast Dietary Supplement Affects Rumen Bacterial Population Dynamics and Fermentation Parameters of Tibetan Sheep (Ovis aries) in Alpine Meadow
Source: Front Microbiol. 2021 Jul 2;12:663945. doi: 10.3389/fmicb.2021.663945 (PMC8283570; doi:10.3389/fmicb.2021.663945)

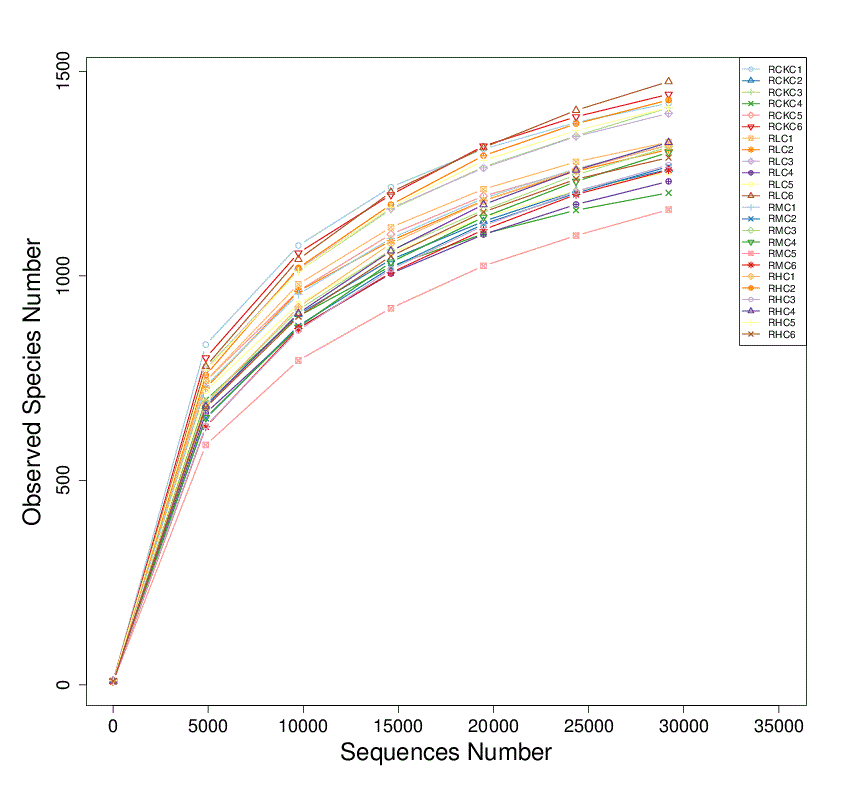

Supplement: Supplementary file 2 [file Image_1.tif]
